# Single session therapy in pediatric healthcare: the value of adopting a strengths-based approach for families living with neurological disorders

**DOI:** 10.1186/s13034-022-00495-6

**Published:** 2022-07-22

**Authors:** Janice Mulligan, Heather Olivieri, Katarina Young, Jia Lin, Samantha J. Anthony

**Affiliations:** 1 Division of Neurology, Gary Hurvitz Centre for Brain and Mental Health, The Hospital for Sick Children, 555 University Avenue, Toronto, ON M5G 1X8 Canada; 2 Department of Social Work, The Hospital for Sick Children, 555 University Avenue, Toronto, ON M5G 1X8 Canada; 3 Child Health Evaluative Sciences, Peter Gilgan Centre for Research and Learning, The Hospital for Sick Children, 686 Bay Street, Toronto, ON M5G 0A4 Canada; 4 Factor-Inwentash Faculty of Social Work, University of Toronto, 246 Bloor Street West, Toronto, ON M5S 1V4 Canada

**Keywords:** Single session therapy, Brief therapy, Narrative therapy, Neurology, Social work

## Abstract

**Background:**

Pediatric patients with neurological disorders often require lifelong management of symptoms and behaviours that can result in enduring emotional burden, stress and impacted health-related quality of life. Single session therapy (SST) draws upon patients’ existing skills and knowledge and has emerged as a therapeutic approach to address pediatric patient and family needs in a timely manner. This study aimed to assess the clinical effectiveness of SST for pediatric patients with neurological disorders and their families, considering self-efficacy, distress, anxiety, therapeutic alliance and client satisfaction, as well as perceptions of whether SST met their pressing needs.

**Methods:**

A convergent parallel mixed-methods design included quantitative data collection via five standardized questionnaires across three time points and qualitative data collection through semi-structured interviews. Quantitative and qualitative data were analyzed independently and then integrated.

**Results:**

The study comprised of 135 participants, including patients, parents and siblings across diverse neurological conditions. Scores of self-efficacy and anxiety in children, and distress and anxiety in adults, improved significantly after the SST. Notably, changes in anxiety in adults remained significant five to seven weeks after the SST. Seventeen participants participated in 12 semi-structured interviews. Participants described that SST (1) was a missing piece in ongoing clinical care, (2) illuminated existing strengths and resilience, and (3) effected a lasting impact beyond the single session.

**Conclusions:**

SST may be a promising initial, strengths-based treatment to support the short-term and potentially long-term needs of pediatric patients with neurological disorders and their families by emphasizing existing strengths, supporting therapeutic alliance and cultivating hope.

**Supplementary Information:**

The online version contains supplementary material available at 10.1186/s13034-022-00495-6.

## Introduction

Single session therapy (SST) has emerged as a promising therapeutic approach to address the psychosocial needs of pediatric patients and their families in a timely manner [1]. SST refers to a single session intervention between client and therapist that is whole and complete, with the provision that additional sessions are available if needed [2, 3]. SST is based on brief, narrative and strengths-based therapeutic approaches that focus on patients’ interests, aspirations, abilities or knowledge to foster one’s ability to thrive in adverse circumstances [4]. Specifically, it emphasizes that: (1) clients have agency and the capacity for change, (2) clients have the ability to generate solutions and new ways of thinking, (3) it is normal for clients to experience challenges in life, (4) clients may not want or need more than one session, (5) supporting small change for clients can lead to larger changes, and (6) collaborative relationships are present between clients and therapists. SST recognizes that clients may need brief therapeutic support during certain periods in their lives, and that a single session can address immediate concerns and potentially improve psychosocial outcomes [2].

While there has been an increase in the delivery of SST, existing research has predominantly characterized and evaluated SST within community health settings, such as mental health walk-in clinics and family health and community-based mental health centres [2, 5–8]. Results from these studies suggest that SST may lead to improvements in self-reported or perceived levels of depression, anxiety, distress and parenting skills [6]. These findings are significant as clients’ perceived mental health is associated with mental health service usage, and thus represents a tangible and important outcome in evaluating mental health interventions [9]. High client satisfaction rates have also been reported following SST, as well as an appreciation for the accessibility of care and team approach [10]. Aside from evaluation within community health settings, there is a paucity of research evaluating SST within a hospital setting and, more specifically, within pediatric chronic illness populations. Further, methodological limitations of SST evaluation have been identified in research, including a lack of standardized measures, concerns of bias when therapists participate in data collection [11], and the use of small and homogenous samples [6]. Hymmen et al. (2013) recommend that further research should include larger and more diverse sample sets, standardized measurement tools and instruments, as well as qualitative approaches to examine the therapeutic nature of SST [6].

The Neurology Social Work Single Session Clinic (NSWSSC) at The Hospital for Sick Children (SickKids) was piloted in 2013, and social workers began offering SST by appointment to pediatric patients with neurological disorders (ND) and their families. ND are a diverse group of disorders that impact the brain and nervous system, including headache disorders, stroke, epilepsy, neuromuscular disorders and movement disorders. Approximately one in six children have a ND [12] and often show difficulties with speech, motor skills, learning and emotion [13]. While symptoms and behaviours can evolve as patients age, ND often require lifelong management with few treatment options available. This can result in enduring emotional burden for patients and families, leading to psychosocial challenges and impacted health-related quality of life [14].

Effective clinical interventions developed for patients and families living with ND must consider the multitude of factors contributing to patient and family stressors, such as child-specific disease trajectories, physical and mental health, social determinants of health and family trauma history [15, 16]. Further, while patients and families with ND experience chronic, heightened levels of stress, they may also experience periods of adaptive coping when therapeutic support may not be needed [17, 18]. Given this range of psychosocial responses, a tailored and accessible therapeutic intervention is essential. As a new approach to clinical practice, SST may be particularly well-suited for patients and families with ND to address the unpredictable and shifting nature of living with a ND.

The NSWSSC, to our knowledge, is the only clinic to offer SST within a pediatric hospital setting. The purpose of this mixed-method study was to assess the clinical effectiveness of SST for pediatric patients with ND and their families, considering self-efficacy, distress, anxiety, therapeutic alliance and client satisfaction, as well as to understand patients’ and families’ perceptions of whether SST met their current needs. The following two research questions were addressed: (1) Does SST, as an intervention that integrates brief and narrative therapeutic models, impact self-efficacy, distress, anxiety, therapeutic alliance and client satisfaction in pediatric patients with ND and their families? and (2) What are the experiences of patients and families who use SST and their perceptions about whether SST met their needs?

## Materials and methods

### Setting and design

The current study was associated with the NSWSSC, a part of the Division of Neurology and the Gary Hurvitz Centre for Brain and Mental Health at SickKids. A convergent parallel mixed-methods design was selected purposively to allow for an in-depth understanding of the research topic as informed by both quantitative and qualitative data [19] (Table 1). More specifically, this approach guided quantitative and qualitative data sets to be explored independently, yet analyzed as an integrated whole [20] when evaluating SST for pediatric patients and families with ND. Standardized questionnaires were administered to participants at repeated intervals, immediately before SST (T1), immediately after SST (T2), and, five to seven weeks after SST (T3) to determine intervention effectiveness on self-efficacy, distress and state anxiety. Questionnaires were also administered at T2 to evaluate therapeutic alliance and client satisfaction. Semi-structured interviews were conducted with a subsample of participants at T3 to explore their experiences and perspectives participating in SST. Institutional Research Ethics was approved by SickKids (#1000055320).

**Table 1:** Mixed-methods design

	Pre-session (T1)	Post-session (T2)	Five to seven weeks post-session (T3)
NIH toolbox self-efficacy survey	X	X	X
Distress thermometer	X	X	X
State trait anxiety inventory	X	X	X
Working alliance inventory-short revised		X	
Client satisfaction questionnaire		X	
Semi-structured interviews			X

### Participants

Participants represented a convenience sample of patients with ND and their families who attended the NSWSSC. Participants met eligibility criteria if they were (i) scheduled for SST, (ii) eight years of age or older, and (iii) English-speaking. Participants with mental or cognitive disorders or who would experience undue hardship from participation were excluded from the study. This decision was determined by the healthcare provider most knowledgeable about the family. Participants were invited to participate in the study via phone. Prior to their SST, participants provided written informed consent or assent. Informed consent was also obtained from a parent/legal guardian if a child provided assent. Each participating family completed a demographics form. Medical data, including diagnosis, date of diagnosis, treatment regimen and complications, were obtained from participating patients’ health records.

### Questionnaires

Five widely-used and validated standardized questionnaires were administered electronically to each participant.

- 1. The NIH Toolbox Self-Efficacy Survey (NIHSE) is a 10-item questionnaire assessing self-efficacy, including one’s ability to manage daily stressors and control over meaningful events [21]. Responses are scored using a 5-point scale and higher scores indicate higher levels of self-efficacy. The NIHSE has shown strong psychometric properties in the areas of dimensionality and precision of scores [22].
- 2. The Distress Thermometer (DT) is a single-item visual analogue scale assessing level of distress in the past week, ranging from 0 (“no distress”) to 10 (“extreme distress”) [23]. Within pediatric healthcare settings, the DT is a sensitive measure with demonstrated concurrent validity [24]. A “traffic light system” was used for scoring: 0 to 4 = green, 5 to 6 = yellow and 7 to 10 = red [25].
- 3. The State Trait Anxiety Inventory (STAI) is a 40-item questionnaire assessing state and trait anxiety [26]. In this study, only the 20-item state anxiety subscale was used to examine participants’ subjective feelings of worry, tension and symptoms of the autonomic nervous system. Responses are scored using a 4-point scale and higher scores indicate higher levels of anxiety. Scores equal or greater than 35 for children and 40 for adults were classified as high levels of anxiety. The STAI is reported to have good reliability and moderate validity [27].
- 4. The Working Alliance Inventory-Short Revised (WAI-SR) is a 12-item questionnaire measuring the therapeutic alliance between a client and their therapist based on three domains: goals of treatment, tasks required to achieve treatment goals and quality of the bond established between client and therapist [28]. The WAI-SR is rated on a 5-point Likert scale. Higher scores indicate a stronger therapeutic alliance and total scores above 36 represent “positive alliance” [29]. Reliability and validity for the WAI-SR have been demonstrated in children, adolescent and adult populations [30].
- 5. The Client Satisfaction Questionnaire (CSQ-8) is an eight-item questionnaire exploring overall satisfaction with health-related services, such as quality of service and likelihood of using the service again [31]. Responses are recorded on a 4-point Likert scale and higher scores indicate a greater level of satisfaction. The cut-off scores proposed by Smith et al. (2014) was used for interpretation: 8 to 13 = poor, 14 to 19 = fair, 20 to 25 = good and 26 to 32 = excellent [32]. The CSQ-8 has reported good reliability and construct validity [33] and has been used to assess and measure parental and adolescent satisfaction with pediatric mental health services [34].

### Qualitative interviews

An invitation to participate in a semi-structured interview at T3 was offered to a subsample of participants recruited from the existing convenience sample. Participants were purposively recruited [35] across age, gender, diagnosis and length of time involved with the NSWSSC. Interviews were guided by interpretive description methodology [36], using an open-ended script that was informed by available literature and the research teams’ clinical expertise (Additional file 1: Appendix S1). An experienced qualitative interviewer, independent of the NSWSSC, conducted face-to-face interviews which lasted approximately 60 to 90 min. All interviews were audio-recorded, transcribed verbatim and de-identified to protect participant confidentiality.

### Quantitative data analysis

Total scores for the NIHSE, DT, and STAI were calculated appropriately. The change in these Total Scores between each time point (T1, T2 and T3) were compared. For participants aged 18 and older (adults), there was no grouping variable. For participants aged 8 to 17 (children), age was the grouping variable: 8–12 years and 13–17 years, respectively. A mixed-model repeated measures analysis was used to account for the within subject correlation. The overall effect of Time as well as pairwise comparisons were assessed within the context of the repeated measures analysis. Total scores for the WAI-SR and CSQ-8 were calculated to describe the mean and standard deviation. The level of significance was set at 5%. SAS™ v. 9.4 software was used.

### Qualitative data analysis

Qualitative analysis was informed by interpretive description which is rooted in an interpretive framework that understands human experience as subjective, contextual, and socially constructed [36]. This approach was selected to allow for the development of evidence-based knowledge grounded in participants’ experiences and perspectives with aims to advance clinical practice [36]. Data analysis followed an evolving, inductive, and systematic process that began with immersion in the data. Interview transcripts were independently coded by two researchers and subject to constant comparative analysis [37]. The research team reviewed codes for consensus and then condensed codes into categories before identifying emergent themes. Team members further compared and contrasted findings between similar and different participant categories to broaden thematic understanding [37]. Trustworthiness was addressed through methods of prolonged engagement, peer debriefing, and member checking before team members reached consensus on key themes that described participants’ experiences with SST [38, 39]. The data management program N-Vivo was used [40].

## Results

The study included 135 participants; all participants completed T1 and T2 and 82 (61%) completed T3. The distribution of participant groups (e.g., mothers, fathers, siblings and patients) at T1 and T3 is provided in Table 2. Overall, mothers represented the majority of study participants at both T1 and T3, followed by patients, fathers, and siblings. At both time points, the sample comprised predominantly of adult participants (~ 70%), ages 18 or older. Children, ages 8 to17, comprised approximately 30% of the participant population. There was no evidence of a statistical or clinical difference between participant groups across the time points. Epilepsy or seizure was the most common primary patient diagnosis for participants.

**Table 2:** Participant demographics

	T1 (n = 135)	T3 (n = 82)	SSI (n = 17)
Participant type, n (%)			
Mother	66 (48.9)	45 (54.9)	10 (58.8)
Father	23 (17.0)	12 (14.6)	1 (5.9)
Patient	37 (27.4)	21 (25.6)	6 (35.3)
Sibling	7 (5.2)	4 (4.9)	–
Legal guardian	1 (0.7)	–	–
Extended family	1 (0.7)	–	–
Age range, n (%)			
8 to 12 years	13 (9.6)	6 (7.3)	2 (11.8)
13 to 17 years	30 (22.2)	19 (23.2)	4 (23.5)
18 + years	92 (68.1)	57 (69.5)	11 (64.7)
Sex, n (%)			
Female	97 (71.9)	61 (74.4)	14 (82.4)
Male	38 (28.1)	21 (25.6)	3 (17.6)
Patient primary diagnosis, n (%)			
Epilepsy/seizure	39 (28.9)	16 (19.5)	4 (23.5)
Headache disorders	11 (8.1)	5 (6.1)	2 (11.8)
Stroke	7 (5.2)	6 (7.3)	–
Adolescent idiopathic scoliosis	6 (4.4)	5 (6.1)	4 (23.5)
Vision disorders	5 (3.7)	3 (3.7)	1 (5.9)
Multiple sclerosis	4 (3.0)	4 (4.9)	2 (11.8)
Global developmental delay	3 (2.2)	1 (1.2)	–
Brain injury	2 (1.5)	2 (2.4)	–
Other	13 (9.6)	10 (12.2)	1 (5.9)
No diagnosis listed	45 (33.3)	30 (36.6)	3 (17.6)
Date of primary diagnosis, n (%)			
2016–2017	5 (3.7)	4 (4.8)	4 (23.6)
2018–2019	77 (57.0)	47 (57.2)	10 (58.8)
Not listed	53 (39.3)	31 (37.8)	3 (17.6)
Past NSWSSC visits, n (%)			
0	119 (88.1)	74 (90.2)	16 (94.1)
1–2	14 (10.4)	6 (7.3)	1 (5.9)
3 +	2 (1.5)	2 (2.4)	–

### Quantitative findings

Descriptive results of the standardized questionnaires across the repeated intervals are presented in Table 3. Results from the mixed-model repeated measures analysis are displayed in Table 4. Compared to baseline, children (ages 8 to 17) reported a significantly higher mean self-efficacy (NIHSE) score at T2. Children also reported a significantly lower mean state anxiety (STAI-C) score at T2. There were no significant differences in distress (DT) scores across the three time points. For children, an Age-Time interaction was entered into the regression model. This interaction was subsequently removed since the effect size was assessed not to be clinically significant. The QQ plots of residuals showed no evidence of departure from normality, validating the assumption of the model.

**Table 3:** Standardized questionnaire results

Questionnaire	T1 Mean (SD)	T2 Mean (SD)	T3 Mean (SD)
Children ages 8 to 17 (n = 43)			
NIHSE	35.42 (6.93)	37.40 (7.29)	35.83 (6.29)
DT	4.307 (2.598)	3.640 (2.636)	4.965 (2.648)
STAI-C	33.86 (7.43)	30.62 (5.83)	35.77 (7.07)
Adults ages 18 + (n = 92)			
NIHSE	29.53 (6.03)	30.03 (5.76)	28.19 (5.88)
DT	5.859 (2.586)	5.167 (2.522)	5.777 (2.376)
STAI-A	42.14 (11.94)	35.95 (10.14)	27.64 (21.58)
Ages 8 to 18 + (n = 135)			
WAI-SR total	–	35.84 (4.55)	–
WAI domain: Goal	–	9.72 (2.35)	–
WAI domain: Task	–	12.92 (1.79)	–
WAI domain: Bond	–	13.85 (2.38)	–
CSQ-8	–	24.49 (2.02)	–

**Table 4:** Mixed-model repeated measures analysis

Questionnaire	Difference Time 1 and time 2 (CI)	Difference Time 1 and time 3 (CI)	p-value
Children ages 8 to 17 (n = 43)			
Total NIHSE time only	1.976 (0.557, 3.339)	1.255 (− 1.200, 3.711)	0.0267
DT	− 0.667 (− 1.516, 0.181)	0.331 (− 0.734, 1.395)	0.1239
Total STAI-C time only	− 3.238 (− 5.354, − 1.121)	0.712 (− 1.970, 3.396)	0.0028
Adults ages 18 + (n = 92)			
Total NIHSE time only	0.500 (1.464, − 0.464)	− 1.710 (− 2.856, − 0.563)	0.0008
DT	− 0.691 (− 1.105, − 0.276)	− 0.195 (− 0.691, 0.301)	0.0043
Total STAI-A time only	− 6.195 (− 9.625, − 2.765)	− 14.612 (− 18.079, − 11.145)	0.0001

In adults (ages 18 and older), mean state anxiety (STAI-A) scores significantly declined across the three time points. Adults reported a high mean state anxiety score at T1 (M = 42.14; SD = 11.94), which decreased significantly at T2 (M = 35.95; SD = 10.14) and T3 (M = 27.64; SD = 21.58). Adults also reported a significantly lower mean distress (DT) score at T2. Conversely, mean self-efficacy (NIHSE) scores significantly decreased at T3.

Overall, participants rated their client satisfaction (CSQ-8) with SST as “good” (M = 24.49; SD = 2.02) and the mean therapeutic alliance (WAI) score was 35.84 (SD = 4.55), indicating “fairly often” positive interactions with their social worker. The strongest alliance was reported in the domain of quality of bond established between participant and social worker.

### Qualitative findings

Twelve semi-structured interviews were conducted with a subsample of 17 participants to capture their experiences and perspectives of SST, comprising 10 mothers (59%), 1 father (6%), and 6 patients (35%). Two patients were aged 8 to 12 and four patients were aged 13 to 17 (Table 2). Five interviews consisted of patient and parent dyads. For most participants (94%), the study session represented their first time attending SST. Three themes emerged from data analysis, as described in detail below (Table 5).

1. *SST offers a missing piece in ongoing clinical care*
  Following SST, many participants agreed that the therapeutic relationship with their social worker was unique and something that was not always present in encounters with other healthcare providers. Overall, there was a sense that SST was a ‘missing piece’ in addressing the psychosocial needs of pediatric patients with ND and their families. One participant summarized: “*I didn’t realize that’s what I wanted until we were in that session. As it kept going, I’m like ‘oh, this is good’. This is what I actually was looking for but, didn’t know… [the] last piece of the puzzle*” (P-11). Many participants noted the collaborative approach with the social worker seemed like a refreshing change from traditional one-directional healthcare provider led medical assessments: “*It was a lot different. It wasn’t someone just looking at a chart. It was more face-to-face*” (P-12). Also important to participants was the medical knowledge that social workers held about ND, which fostered a tailored approach and made SST distinctive from standard community-based counselling services. One parent shared: “*The fact that they… specialize in neurology… they understood what I was talking about*” (P-1). Overall, participants emphasized the role of SST as an adjunct to other healthcare interventions received at the hospital: “*It really does complete the puzzle for other services that we receive for our daughter*” (P-10).
2. *SST illuminates existing strengths and resilience*
  Using SST as a strengths-based approach to therapy empowered patients and families to identify and expand upon their existing strengths and resilience. Many participants highlighted the supportive role of the social worker in nurturing self-determination in ways that felt validating: “*To… feel validated and feel that positivity that we were on the right track*” (P-5). Some participants acknowledged how specific questions led to them noticing existing skills: “*You can see your skills because of the questions asked*” (P-2). Recognizing previously successful approaches to problem-solving also provided participants with validation about their internal strengths and encouraged many to apply these skills to their current concerns: “*Focusing on those skills again… I had the power of getting things solved*” (P-2).
  In addition to identifying existing skills, the collaborative nature of the SST enabled participants to pinpoint pressing needs with a focus on small and attainable goals. This encouraged many participants to envision and pursue their goals with increasing momentum and motivation. One participant described: “*[SST] gave me… parameters or structures to work on… I was progressing*” (P-8). Another participant shared: “*I felt like I had taken a step forward*” (P-1). Following SST, many participants expressed feelings of increased support and connection from the therapeutic encounter: “*I didn’t feel like all of the weight was on me. There were other people who could help me carry that load*” (P-1). This was also described by a parent who reported needing reminders of their own strengths, creating space for alternative narratives: “*Somebody should remind you… to see [the] strong side of yourself*” (P-2). Many participants experienced an enhanced appreciation of their inherent resilience and felt empowered to utilize their existing skills moving forward: “*I left there focusing on my skills and focusing on giving more compassion to myself*” (P-2).
3. *SST has a lasting impact beyond the single session*
  Through patient and family narratives, it was clear that the impact of SST lasted for participants beyond the session itself. Specifically, participants described remembering their session and applying learned skills and strategies into their everyday lives: “*Time to time I go back and see what the questions were and reflecting those questions to myself again*” (P-2). Another participant said: “*We started to figure out, figure things out more… [SST] was a factor… a very helpful one*” (P-16). Participants highlighted how the strengths-based approach encouraged a future-focused view that increased their motivation to continue working towards their goals. However, participants also acknowledged the ongoing challenges that persisted due to living with ND. In this context, SST did not “fix” their concerns, but provided them with a renewed perspective and alternative coping strategies to “*keep working at it*” (P-10). One participant said: “*I don’t think it’s 100% we’re there, but I think that’s life*” (P-7). As new challenges emerged, participants maintained strength and resilience to cope “*months and weeks after*” (P-12).
  Associated with the long-term benefits participants attributed to SST, participants described an enhanced sense of hope and ability to “*see the brighter side*” (P-3). This was especially striking given the multitude of stressors facing patients and families living with ND. Many participants described this shift in mindset in transformative ways: “*Turning... that darkness and that hopelessness into something positive and something productive*” (P-1); “You can feel that hope and feel that lightness again” (P-1). Hope was connected to SST as many participants were hopeful about their ability to leverage their strengths in the face of unpredictability: “*You’re always going to have bad days or things that happen that you don’t want but, there’s always good that comes after it*” (P-7).

**Table 5:** Qualitative themes

Theme	Sub-theme	Quotations
SST was a missing piece in clinical care	Missing piece	• “I didn’t realize that’s what I wanted until we were in that session. As it kept going, I’m like 'oh, this is good'. This is what I actually was looking for but, didn’t know that was what I was looking for… last piece of the puzzle.” (P-11) • “It felt great because it felt like it was completing the whole package. I think a lot of people don’t understand that yes, a child is ill but, they have caregivers too, who are important in order to get this child on the best path.” (P-10) • “I think it really seems to fit a gap, that I'm not sure exactly how it would have been filled otherwise.” (P-16) • “It really does complete the puzzle for other services that we receive for our daughter.” (P-10)
	Individualized approach	• “It was a lot different. It wasn’t someone just looking at a chart. It was more face-to-face.” (P-12) • “Different, because it was focusing on me. It was nice to look at my particular situation, so tailored.” (P-1) • “It was good to have someone too, who is… interested in… emotional concerns… And who you could talk to… at more length, not just about medical issues.” (P-16) • “[SST] is good because you are able to focus more on yourself… instead of focusing on a certain part of your life like other departments may do.” (P-17)
	Medical knowledge	• “The other thing too that I found that was really helpful for the Single Session Clinics is the fact that they had, that because they specialize in neurology… they understood what I was talking about.” (P-1) • “Just the fact that they… work in that particular setting did give me some… extra trust and comfort.” (P-8)
SST illuminated existing strengths and skills	Validating skills	• “To go and feel validated and feel that positivity that we were on the right track was really helpful for us.” (P-5) • “You can see your skills because of the questions asked.” (P-2) • “When we came to the session it was good because you guys had key questions that were prompting, which was awesome. It did trigger some things that [the patient] did get to speak about. So, that was the good part of going there.” (P-11) • “Yeah, it [reminded me of my]… skills of life… and focusing on those skills again. […] I had the opportunities, and I had the power of getting things solved. […] I left there focusing on my skills and focusing on giving more compassion to myself.” (P-2)
	Process over perfection	• “[SST] gave me other parameters or structures to work on that I could actually keep myself busy doing and feel like I was progressing or like I was continuing to do things for us.” (P-8) • “I felt that I had taken a step forward.” (P-1) • “They spoke about what to do in the moment. So, I thought that was helpful.” (P-3) • “What we actually got was more practical help with coordinating resources for caring – things like that. That was very, very useful.” (P-16)
	Support and connectedness	• “I didn’t feel like all of the weight was on me. There were other people who could help me carry that load.” (P-1) • “It was helpful to get someone to talk to and to vent.” (P-15) • “I just didn’t feel so alone. […] It felt like they were taking action right away.” (P-1) • “Somebody should remind you… you need some help to see your strong side of yourself.” (P2) • “It’s so good to know that there is someone… there who cares for me so that I can care and advocate for my daughter. […] That someone was advocating on my behalf, and [helped] me to advocate for my daughter.” (P-1)
SST effected a lasting impact	Lasting impact	• “Time to time I go back and see what the questions were and reflecting those questions to myself again.” (P-2) • “It can give you some good tips for even when you leave. And for the following months and weeks after.” (P12) • “Long lasting effect… after a while, we just had more time now – we started to figure out, figure things out more… [SST] was a factor but, it was a very helpful one.” (P-16) • “You just don’t realize something has impacted you so much until you really look back on it.” (P-1)
	Ongoing challenges	• “I have to keep working at it.” (P-10) • “I don’t think it’s 100% we’re there, but I think that’s life.” (P-7) • “I still think it’s a work in progress.” (P-3) • “I think it was mostly a shift in the mindset… Like acknowledging it and just kind of accepting it.” (P-10)
	Enhanced hope	• “The brighter side of the situation.” (P-3) • “Turning that, that darkness and that hopelessness into something positive and something productive.” (P-1) • “I think I was less distressed for sure. I felt like – like not like ‘oh everything’s great,’ but, it was like okay – again that ah mention of hope. […] I think hopeful that we had taken a step, that things were starting to look better and that hopefully that they would continue.” (P-7) • “To put that worry into something that’s you know – that you can feel that hope and that lightness again.” (P-1) • “That reminder that you’re always going to have bad days or things that happen that you don’t want but, there’s always good that comes after it.” (P-7)

## Discussion

The purpose of the current study was to evaluate the clinical effectiveness of SST for patients and families living with ND and to explore participants’ perceptions about whether their involvement in SST met their needs. To our knowledge, this is the first study to examine SST within a pediatric hospital setting and contributes to much-needed empirical literature exploring the implementation and evaluation of SST within pediatric healthcare.

In the current study, participants’ client satisfaction with SST, as measured using the CSQ-8, was reported as “good”, similar to scores in previous SST research within mental health settings [2]. Participants’ interview responses supported questionnaire findings as the majority of participants reported a positive experience with SST. Collectively, participants characterized SST as an aspect of care that was a ‘missing piece’ in their usual clinical encounters. More specifically, participants described appreciating the tailored approach of SST for their personal experience living with a ND as well as the collaborative nature of their social worker. Recent research identifies that the most helpful aspects of SST, as rated by family members, are the customized advice and expertise offered by their therapist in addition to increased support and validation. [41]. Additionally, Westwater et al. (2020) identified themes relating to collaboration as particularly helpful to clients attending SST, including creating a space for communication and feeling validated [42]. Similarly, in the present study, therapeutic alliance, as defined as the positive “interactive, collaborative elements” between patient and therapist [43] and measured using the WAI, was found to occur “fairly often”, especially in the domain of “quality of bond established within the therapeutic relationship”. This finding is consistent with research suggesting that one session can be enough to foster strong therapeutic alliance [44, 45].

Of importance, research suggests that therapeutic alliance is a significant factor for improved outcomes in therapy [46]. In the current study, questionnaire findings indicated improved outcomes in self-efficacy and state anxiety in children, and distress and state anxiety in adults, both immediately following SST. Notably, improvements in adult state anxiety remained significant after five to seven weeks, representing a clinically meaningful and sustained change [26]. This finding is important as reduced anxiety in caregivers has been found to mitigate levels of anxiety in children with chronic illness [47]. Reduced anxiety can promote optimal coping behaviours in the face of chronic illness and create space for alternate stories to blossom. Research suggests that the benefit of these stories is the “anti-problem” focus that shines a light on competencies, abilities and values [48].

Despite significant improvements in adult state anxiety, questionnaire findings in the current study did not suggest that SST had a lasting impact on child state anxiety and self-efficacy or distress in either children or adults. Considering the concept of chronic sorrow may be helpful in interpreting these mixed results. Living with a ND is unpredictable with cyclical periods of sorrow, joy and fluctuating emotional responses [49, 50]. Since chronic sorrow often accompanies chronic illness [51, 52], it may be unreasonable to expect long-term improvements to be evident using quantitative measures alone as they often limit nuanced explanations and interpretations of coping.

Participant interview findings suggested that patients and families living with ND did experience longer term benefits from SST, which challenges the notion that lasting change can only be made through long-term interventions [53]. These findings support previous results that showed a significant increase in clients’ self-perceived coping six weeks after SST [11]. Participants in the current study described reflecting on their session at a later time and implementing new or realized strategies to cope with events in their lives. In addition, participants described feeling empowered to use their existing strengths and the skills that their social worker had helped them to recognize. Gaining new perspectives on a problem and generating novel solutions has been cited as a beneficial strategy for SST clients [42]. Patients and families in the current study were empowered to continue implementing learned strategies following the SST, even over time, supporting the view that SST continues to activate therapeutic processes post SST [6].

Our qualitative findings further revealed an enhanced sense of hope among interview participants. Specifically, participants identified that a sense of hope was cultivated from social worker-participant interactions which offered support as they navigated the complexities of their lives. Instilling hope has been identified as a powerful tool for fostering well-being and resilience for patients and families negotiating the uncertainty associated with chronic illness [54]. A SST approach is inherently hopeful as it uses language and questions that emphasize options, possibilities and future-focused orientation, all while highlighting clients’ agency [55, 56]. In an evaluation of a walk-in SST, adult clients reported increased levels of hope after SST [7]. These changes were maintained during the one-month follow-up with additional improvements in mental health and coping [7]. Snyder’s Hope Theory describes hope as an action-oriented process, whereby an individual has the strategies needed to achieve one’s goals and a belief and motivation that goals are achievable [57]. In the current study, participants highlighted the following strategies as learned aspects of SST: an increased appreciation of strengths-based skills, a shift in mindset to positive thinking, and the ability to identify and apply existing skills to new problems. These findings may provide insight to understanding the observed association between SST and increased hope in patients and families.

### Limitations

Only English-speaking participants were included in the study, and thus, our study sample may not be representative of the overall diverse ND population. Additionally, there was loss to follow up at T3 despite multiple efforts to remind participants to complete measures. This may have impacted the repeated measures analysis; however, 17 participants shared their experiences through qualitative interviews at the T3 timepoint which also captured changes over time.

## Clinical relevance

Our findings contribute to the existing literature that views SST as an effective intervention approach to emphasize existing strengths and coping strategies, and specifically supports using SST for patients and families with ND. Strengths-based approaches can enhance hope and bring forward capacity and competencies that assist with buffering stress, while also highlighting opportunities for growth [58, 59]. It is possible that understanding clients’ experiences of strengths-based therapy in the form of SST may be impactful when addressing the psychosocial needs of pediatric patients and families living with chronic conditions.

## Conclusion

Our findings suggest that SST may be a promising initial choice of treatment to support short-term and potentially long-term concerns for patients with ND and their families. More research is warranted to examine the potential long-term influence of using SST in this clinical population. Future research could also explore the specific considerations needed to adapt the structure of SST to other pediatric populations living with chronic illness.

## Supplementary Information

**Additional file 1: Appendix S1.** Qualitive interview guide.

## Data Availability

The quantitative data that support the findings of this study are available from the corresponding author, JM, upon reasonable request. The qualitative data (interview transcripts) cannot be shared.

## Abbreviations

ND: Neurological disorder
NSWSSC: Neurology social work single session clinic
SST: Single session therapy
