# Supplementary material for: Single session therapy in pediatric healthcare: the value of adopting a strengths-based approach for families living with neurological disorders
Source: Child Adolesc Psychiatry Ment Health. 2022 Jul 22;16:59. doi: 10.1186/s13034-022-00495-6 (PMC9308282; doi:10.1186/s13034-022-00495-6)
Supplement: Supplementary file 1 — Additional file 1: Appendix S1. Qualitive interview guide. [file 13034_2022_495_MOESM1_ESM.docx]

**Appendix S1. Qualitive interview guide**

1. What were you kind of hoping to get out of the appointment? What did you want to come away with feeling like?
2. What stood out most for you about the SST appointment?
3. What did you take away from the SST appointment?
